# Supplementary material for: Exploring the experiences and views of doctors working with Artificial Intelligence in English healthcare; a qualitative study
Source: PLoS One. 2023 Mar 2;18(3):e0282415. doi: 10.1371/journal.pone.0282415 (PMC9980725; doi:10.1371/journal.pone.0282415)
Supplement: Attachment — Submitted filename: Response to reviewers.docx [file pone.0282415.s001.docx]

Rebuttal letter- response to reviewers

We are extremely grateful to the reviewers feeling this paper does have merit following major revisions. With the help of these comments, we feel these revisions have improved the focus of this paper, stated the aims of the paper clearer and why ‘doctors working with AI’ are key proponents in this discourse, and therefore warrant this qualitative study.

Below we have made a table, with our response to the reviewers comments and further detail in the following column with the lines numbers (where appropriate) demonstrating where these changes have been made.

| Reviewer One comment | Our response | Page number |
| --- | --- | --- |
| While I realise from line 115ff this is stated (although it only became clear to me once I started reading the analysis) I think you need to make it much clearer your project was looking at experiences of AI clinician developers from the outset. | We agree with this suggestion, and we have reviewed the manuscript so that there is consistency in how we refer to the participants as ‘doctors’ throughout the paper | Throughout |
| However, I think that in addition to making the purpose of the paper less ambiguous up front, it also needs a background that considers the barriers/drivers for those who work in medicine and industry, it seems to me that this in many ways is the bedrock of the paper | We agree with this comment/suggestion, however this will be particularly challenging to resolve as the evidence on this area (barriers and drivers) within the field of medical AI is scarce. As mentioned in the aim of this paper, we feel that this is a justified omission given the lack of evidence. However, we have attempted to make the purpose of the paper less ambiguous in the rewrite of the introduction. We have also included a section about barriers to doctors working with health technology, suggesting there may be parallels | Throughout  Lines 179-187 addressed the limited literature on doctors working in industry with AI  New section line 140-162 |
| . In relation to this the discussion really just reiterates the findings, it does not really discuss the very interesting data in light of the literature to any degree. I would have liked to see more literature cited regarding clinical industrial collaboration and moving across this boundary | We thank the reviewer for these comments, and we agree that the discussion was not detailed enough.  However, regarding clinical industrial collaboration and moving across this boundary, we performed a search on pubmed and medline with terms ‘doctor’, ‘clinician’, ‘health tech’, ‘med tech’, ‘industry’, ‘experience’, ‘qualitative’ and did this not yield any relevant results. Therefore, we could not cite more literature regarding this point |  |
| Maybe explain what Topol review is to the uninitiated. | We agree, and we have included a short sentence that explains the review | Lines 89-96 |
| Re ethics : I think it might be advisable to roughen the data in table one a little clearly there may be only one or two ear nose and throat surgeons who are working in the tech sector in the UK, so maybe just call them surgeon, unless the speciality is really important for subsequent points you are making (I don’t think it is) , same for other types of physicians | We agree with this comment and we have amended the table so that references to specialties have been removed | Table 2, between line 223 and 224 |
| Is surely the wrong way around you discuss findings of theme 3 first, but the bulk of the paper is about the ways in which these individuals moved into industry/industrial collaborations and how they experience this. As written the discussion of this aspect of the data comes after the theme three discussion, i.e how they perceive clinical colleagues will perceive and cope with this technology and what sort of tech training they need. | We agree with the comments about the order of the discussion and have amended accordingly.  We have also included limitations and future areas of research. | discussion |
| some typos… Line 59 word missing; studies? Line 86 sentence starting whilst may be missing words Line 234 – system level barriers to what, implementation, working with AI – please expand 362 I assume you mean the concept of “AI” was used …… | We thanks the reviewers for bringing these typos to our attention. | Throughout  Lines 74- have added word ‘studies’ after research  Lines 105- amended the sentence which seemed to be missing words  Lines 317-318- have expanded on system-level barriers  Lines 445- added ‘concept of AI’ |
| Reviewer 2 comments |  |  |
| . However, I found that the introduction/literature review section would benefit from a revision. It could be a lot more focused, narrowed down, with clear definitions of terms and a clear outline and justification of the objectives of the research. | We agree, and we have made the aims and objectives of the paper explicit. We have also reworked the majority of the introduction. | Lines 189-198- objectives and research aims stated |
| First, the introduction does not lay out clearly and specifically what the empirics section is trying to answer. The research aim is vague, and the introduction does not clarify the aims adequately. | We agree with this comment, and this has also been identified by reviewer 1. We have revised the introduction so that the aims and objectives are explicit. We have also reworked the majority of the introduction. | Lines 189-198- objectives and research aims stated |
| Several expressions are being used to refer to clinicians interacting with AI in some capacity: • Engagement with AI (p.1, line 18 and 20) • Doctors working with AI within the NHS (p.2, line 24) • How engagement with AI may increase (p.2, line 26) | We agree with these comments, and we have ensured consistency and now refer to participants as ‘doctors’.  Literature has used the term engagement without clarifying what this means, hence why that term is used in the introduction.  We have clarified what ‘engagement’ means in our study and what ‘working with AI’ means in our study. | Clarification of:  Engagement- lines 136-141  Working with AI- Lines 190-194 |
| Secondly, and related to the first point, instead of explaining at length in the third paragraph how COVID-19 impacted AI development and adoption, the paper could focus more on the literature addressing why AI adoption in healthcare is slow and limited, and single out and explain why the lack of involvement of doctors in the industry is a key determinant. | We appreciate the reviewers comments and agree this does not add to our research question and aims, therefore we have removed mention of COVID-19 and instead added sections regarding literature that may have parallels to doctors and AI: such as doctors barriers to adoption of medical technology, more literature on awareness of doctors with AI. | Introduction |
| Ultimately, there is a lack of a justification for why the authors decide to interview this group of people specifically. For example, why not clinicians working with AI in their everyday practice? If the argument is that NHS staff should be involved with AI in order to, in turn, persuade colleagues, clinicians who use AI tools are arguably also well placed to persuade their colleagues that technology-supported service is safe. | We agree with this comment. We have even made reference to colleagues using medical technology as being key agents in persuading other colleagues that the medical technology is acceptable (line 99-115). We have made it clearer throughout the introduction that AI is in its infancy and is not at widespread stage of adoption in clinical use, therefore there are not doctors in the NHS that are using AI tools in their clinical practice. Therefore, we have chosen to focus on doctors developing AI. | Throughout introduction, but following lines in particular focus on addressing this reviewer comment  Lines 138-141  Lines 192-194  Lines 202-208 |
| • Page 5 line 102: it is not immediately obvious how the engagement and education of healthcare professionals supports Greenhalgh et al’s theory (there is a typo in her name, no "u"). It might be helpful to add an explanatory sentence. • Page 13 line 210: the paragraph about work-life balance does not seem directly relevant to the title of the sub-section “the route to working with AI”. This should perhaps become a different/additional section. | Thank you for highlighting these comments, we have addressed them | We have reworked paragraphs to explain how the engagement and education of healthcare professionals support Greenhalgh’s theory within Lines 117-134  We feel work-life balance still belongs in this section, we have conveyed clearer that work-life balance is a factor which keeps doctors working with AI, rather than reverting back to full-time clinical roles within lines 292-298 |
